# Notes and Notices

**Published:** 1892-10

**Authors:** 


### NOTES AND NOTICES.

IN CONVALESCENCE.—The lack of suitable foods for convalescents from severe illness, and in the treatment of typhoid and other low fevers is often felt by the practical physician. Milk while of very great use, often cannot be taken, and often causes trouble on account of the indigestibility of the casein. The various beef extracts are more stimulating than nourishing, and the majority of the prepared foods offered are either unpleasant to the taste or difficult of digestion, and unsuited to the needs of the case.

In such cases Malted Milk will form a very welcome addition to the dietary of the sick-room. The basis of this food is pure, fresh, sterilized milk, in which the casein is rendered digestible by the action of the plant pepsin produced by a special method of malting the cereals originated by the manufacturers. It is pleasant to the taste, simply prepared by dissolving in water, no cooking required, and will be retained and assimilated in many cases when all other foods fail.

MALTED MILK.—In all cases of cholera infantum and summer complaints of children physicians will find a most valuable aid in Horlick's Malted Milk. This infant food is meeting with a very deserved success, being in general use all over the country by physicians and in most of the large asylums and hospitals for children, and giving uniform good results. It is frequently retained and assimilated in difficult cases where all other forms of nourishment are rejected. It contains no starch and will not coagulate in the stomach like raw milk. Try it in your next case of infantile trouble and mark results. All druggists keep it.

DR. H. S. PHILLIPS has removed to No. 73 Congress Street, Pittsburgh, Pa.

DR. MARY FLORENCE TAFT has removed from Waterbury, Conn., to 271 Oakwood Boulevard, Chicago.

A GOOD OPENING FOR A PHYSICIAN—young, unmarried, and a homœopathist, in Texas. The field was occupied by a young man until last spring, when he was compelled to leave on account of his health. The people are very anxious for a homœopathist to come among them. For particulars address Drs. Thatcher & Thatcher, Dallas, Texas.

